# Pseudo-Sanger sequencing: massively parallel production of long and near error-free reads using NGS technology

**DOI:** 10.1186/1471-2164-14-711

**Published:** 2013-10-17

**Authors:** Jue Ruan, Lan Jiang, Zechen Chong, Qiang Gong, Heng Li, Chunyan Li, Yong Tao, Caihong Zheng, Weiwei Zhai, David Turissini, Charles H Cannon, Xuemei Lu, Chung-I Wu

**Affiliations:** Laboratory of Disease Genomics and Individualized Medicine, Beijing Institute of Genomics, Chinese Academy of Sciences, Beijing, 100101 People’s Republic of China; Broad Institute of Harvard and MIT, 02142 Cambridge, Massachusetts USA; Department of Ecology and Evolution, University of Chicago, 60637 Chicago, IL USA; Ecological Evolution Group, Xishuangbanna Tropical Botanic Garden, Chinese Academy of Sciences, Menglun, Mengla, Yunnan 666303 People’s Republic of China; Department of Biological Sciences, Texas Tech University, 79409 Lubbock, TX USA

**Keywords:** Next-generation sequencing, Gap filling, Genome assembly

## Abstract

**Background:**

Usually, next generation sequencing (NGS) technology has the property of ultra-high throughput but the read length is remarkably short compared to conventional Sanger sequencing. Paired-end NGS could computationally extend the read length but with a lot of practical inconvenience because of the inherent gaps. Now that Illumina paired-end sequencing has the ability of read both ends from 600 bp or even 800 bp DNA fragments, how to fill in the gaps between paired ends to produce accurate long reads is intriguing but challenging.

**Results:**

We have developed a new technology, referred to as pseudo-Sanger (PS) sequencing. It tries to fill in the gaps between paired ends and could generate near error-free sequences equivalent to the conventional Sanger reads in length but with the high throughput of the Next Generation Sequencing. The major novelty of PS method lies on that the gap filling is based on local assembly of paired-end reads which have overlaps with at either end. Thus, we are able to fill in the gaps in repetitive genomic region correctly. The PS sequencing starts with short reads from NGS platforms, using a series of paired-end libraries of stepwise decreasing insert sizes. A computational method is introduced to transform these special paired-end reads into long and near error-free PS sequences, which correspond in length to those with the largest insert sizes. The PS construction has 3 advantages over untransformed reads: gap filling, error correction and heterozygote tolerance. Among the many applications of the PS construction is de novo genome assembly, which we tested in this study. Assembly of PS reads from a non-isogenic strain of *Drosophila melanogaster* yields an N50 contig of 190 kb, a 5 fold improvement over the existing de novo assembly methods and a 3 fold advantage over the assembly of long reads from 454 sequencing.

**Conclusions:**

Our method generated near error-free long reads from NGS paired-end sequencing. We demonstrated that de novo assembly could benefit a lot from these Sanger-like reads. Besides, the characteristic of the long reads could be applied to such applications as structural variations detection and metagenomics.

**Electronic supplementary material:**

The online version of this article (doi:10.1186/1471-2164-14-711) contains supplementary material, which is available to authorized users.

## Background

The next generation sequencing (NGS) technology has scaled up DNA sequence acquisition by several orders of magnitude [1, 2]. However, the short read sequences (SRS) from NGS, generally 100 bp or so in length, have only limited uses without further bioinformatic processing [3, 4]. Sequences obtained by the conventional Sanger sequencing methods, generally >600 bp in length, are much more useful but the throughput is too low and the cost is too high. Therefore, an efficient method for increasing the read length from NGS should be valuable.

A major advance in NGS is the development of paired-end (PE) library construction, which generates two short reads from a single DNA fragment separated by an insert of a known size. In principle, longer sequences could be produced post-hoc, if the gap between the paired-ends could be filled correctly. Several attempts have been made to extend the length of short reads by merging the paired-end reads from small fragments into longer single end reads [5–7] and proved the advantages of longer reads in metagenomics and genome assembly. However, due to the requirement of library insert size less than twice of read length, merging of overlapped reads could only increase the read length by a small fraction. The merged reads are often less than doubling the read length. *GapFiller* tried to fill the gaps but not repetitive sequences within a longer insert based on 'seed-and-extend’ strategy in bacteria genomes [8], but its performance in large genomes might decrease due to the largely existing repetitive sequences. *ALLPATHS*[9] is a standalone genome assembler. It efficiently utilized paired-end information by filling the inner gaps using extension, but also suffered much from extensions from one end to the other end of paired-end reads in global graph of reads overlaps. Successive multiple libraries were used in the long march [10] and *SubAssembly*[11]. They used the paradigm of clustering and local assembly, to avoid the repetitive sequences and computing complex in overlap extension. In general, read pairs from the same DNA fragment were indexed with sophisticated unique tags so that they could be locally assembled. However, the application of these methods to large genomes has two major limitations. First, the experiments are complex and cannot be consistently executed. For example, *SubAssembly* requires the dilution of DNA to obtain a desired number of DNA molecules, but the amount obtained may vary by orders of magnitude. Second, the sequencing costs are equivalent to 454 sequencing, which produces long reads directly without a complicated third-parity library preparation. The goal of this study is to fill in the gap between paired-end reads from large DNA fragments (600 or 800 bp), and produce sequences like Sanger reads even when the sequence of gaps is repetitive.

**Figure 1:**
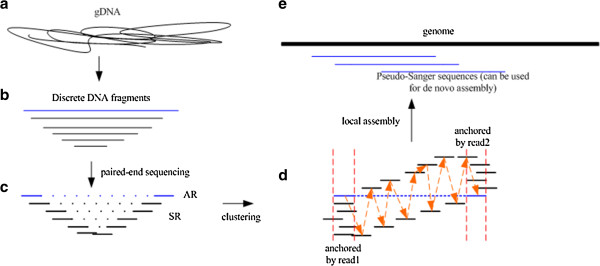
**Construction of pseudo-Sanger sequences.** Genomic DNA **(a)** is randomly sheared into fragments **(b)** of a wide range of sizes. Libraries are constructed for each band with a step size of ~100 bp and subjected to Illumina paired-end sequencing **(c)**. Anchor reads (ARs) come from the library with the largest fragments. The rest are supporting reads (SRs). Each AR is aligned with all SRs that are likely to fill in its internal gap **(d)**. Local assembly is then performed on of the AR plus the associated SRs to construct a pseudo-Sanger sequence. The resulting pseudo-Sanger sequences cover the entire genome much like other WGS reads **(e)**.

We have now developed a new computational approach, referred to as pseudo-Sanger (PS) sequencing (Figure 1), which can generate long reads from paired-end SRS. Unlike previous methods, we sequence successive multiple libraries prepared with standard protocols, take two reads in a pair of large-insert PE reads as a tag, cluster other PE reads that have one end overlapped with it as local reads group, and locally assemble them to fill in the inner gap of the large-insert PE reads. The nested set of libraries are composed of paired-end reads with decreasing insert sizes (e.g. 600 bp, 400 bp, 300 bp, and 200 bp) (Additional file 1: Note S1). The paired-end reads from the library with the largest inserts serve as anchor reads (ARs, Figure 1c). The nested reads with shorter inserts are referred to as supporting reads (SRs, Figure 1c) and are local assembled to fill the gap between the two ARs to create contiguous PS sequences. Because SRs are strongly associated with their AR, the advantage of PS method lies in its local assembly which is less impeded by repetitive sequences. Another advantage of the PS method lies in its operational simplicity and low cost, both only marginally higher than the current practices in generating SRS.

The PS approach is a general computational method that provides the insert sequence between paired-end or mate-pair reads. Since the current end sequences are roughly 100 bp in length, the resulting filled-in sequences happen to approach the length of the conventional Sanger reads. As the paired short reads increase in size, the insert between them can be increased correspondingly and the pseudo-Sanger sequences can be expected to greatly exceed the Sanger reads in length (super-Sanger reads). Besides the increase in read length, PS sequencing corrects most of sequencing errors and tolerates heterozygous sites. With these advantages, PS sequencing can have applications for many problems requiring long and error-free DNA sequences. For example, PS sequences are sufficiently long for the detection of chromosomal structural variations at the base-pair resolution. Furthermore, the analysis of metagenomic diversity by NGS is often hampered by the paucity of species markers due to the absence of long reads, which PS sequencing can rectify. Finally, an obvious application and a stringent test of PS sequencing is whole-genome de novo assembly, which will be reported below.

## Results

### Algorithm for constructing pseudo-Sanger sequences

Our method, implemented in the new software package *anytag*, utilizes a nested set of paired-end libraries with decreasing insert sizes (Figure 1). Three steps are used to construct a pseudo-Sanger sequence for each AR. First, we align ARs against all SRs to obtain candidate SRs located within the span of every AR. Second, we perform a local assembly using these candidate SRs. In general, the local assembly utilizes an overlap-layout-consensus (OLC) approach but with the constraint that the two ends of the layout come from an AR. We refer to the resulting segment as a primitive pseudo-Sanger sequence. Finally, we identify all SRs, both reads of which are located inside the primitive pseudo-Sanger sequence, to call the consensus sequence (pseudo-Sanger sequence).

We have also introduced a module to verify that both reads in an AR are not repetitive sequences. If either read of an AR is deemed to be repetitive, we do not construct a pseudo-Sanger sequence from it.

To efficiently align ARs against SRs, we use block spaced seeds to index the reads of SRs, and scan the AR base by base. When an AR and a SR share the same seed sequence, they are considered a potential match, and the sequence is extended without gaps. If the extension fails due to excessive mismatches, the Smith-Waterman algorithm is used to perform a gap alignment. Otherwise, the result of the simple extension is used as an alignment. If a SR read overlaps with an AR on the same strand (forward overlap), the partners of the SR pair and the AR pair will also have the same orientation. Because we are only concerned with filling the gap between the two members of an AR pair and the distance between any SR pair will always be shorter than the distance between an AR pair, only forward overlapping SR reads and their partners are used in our search. We, therefore, create a localized cluster of SR pairs with the same orientation as the first read of each AR pair, greatly reducing the complexity of the following local assembly.

Our local assembly uses a modified layout step that ensures the final contig starts from one read of AR and ends at the other read. An overlap graph is built with reads as nodes and overlaps as edges. We traverse the graph between the two ends of an AR and try to find a path that connects them. For each end of the AR, a heap table is used to find the path to the other end with the maximum number of overlapped bases. When two traversals meet, we check whether the length of the layout is within the insert range of the AR library.

To improve the quality of the pseudo-Sanger sequences, we add inner SRs (those that map within the span of the AR rather than to the AR) and call the consensus sequence again. To reduce the computing time, we query primitive pseudo-Sanger sequence against all SRs without using gap alignments. If one end of a SR matches, the other end is aligned to the primitive pseudo-Sanger sequence by the Smith-Waterman algorithm.

We calculate the expected number of SRs aligned to a given AR using the formula:

where L is the length of each read, O is the minimum overlap, and D is the sequence depth. ARs with 1.5 times this expected value are considered repetitive sequence and pseudo-Sanger sequences are not constructed for them. To account for potential missing sequence in regions where an AR has been labeled as repetitive sequence and excluded, we iteratively use the next largest insert size library’s paired-end reads as ARs to construct pseudo-Sanger sequences. Additionally, reads that are not used in the local assembly are kept for possible use in closing gaps in the subsequent assembly.

### Construction of pseudo-Sanger sequences from simulated data

The sequenced genome of *Drosophila melanogaster* and Human chromosome 1 were used in our simulation. For each dataset, we simulated 60X genomic coverage with paired-end short reads with a sequencing error of 0.005 and heterozygosity of 0.001. The simulated datasets are composed of four libraries (15× each for the 200 bp, 300 bp, 400 bp and 600 bp libraries).

We used our software *anytag* to convert the simulated paired-end short reads into pseudo-Sanger sequences. In the simulation of *D. melanogaster*, *anytag* generated 22X long sequences, of which the mean length was 614.68 bp. The error rate was reduced from 0.5% to 0.0084%, and was uniformly distributed across the pseudo-Sanger sequences (Additional file 1: Figure S1). In the simulation of Human chromosome 1, *anytag* generated 19X long sequences, of which the mean length was 610.13 bp. The error rate was reduced from 0.5% to 0.021%, and was also uniformly distributed.

### Comparison of genome assemblies from simulated data

Once the pseudo-Sanger sequences were constructed, the *Newbler* program [12] and *minimus2* from the *AMOS* package [13] were used to assemble them into contigs. To compare the effectiveness and accuracy of our method against other current de novo short read algorithms, we chose four general de novo assemblers, *velvet*, *SOAPdenovo*, *ABySS* and *MSR-CA* to directly assemble the same simulated paired-end reads. Each of the four programs can output scaffolds for paired-end short reads, and we treated the continuous sequences (those with no arbitrary bases) in these scaffolds as contigs. To explore the best assemblies for those three programs, we ran them with different parameters and selected the assembly with the largest N50 contig (Additional file 1: Tables S1-S2). The evaluation program from *GAGE*[14] was used to assess the mis-assemblies.

Overall, *anytag* performed substantially better than the other programs (Table 1). *Anytag* always ranked best in N50 contig size (197 k and 106 k), N90 contig size (43 k and 27 k) and mean contig size (66 k and 49 k), for *Drosophila* and human, respectively. For human chromosome 1, which has a larger genome size and is more repetitive, the contig sizes from *anytag* were about three times greater than the best of the other assemblies. *MSR-CA* performed better than the other assemblers (excluding *anytag*). Both *anytag* and *MSR-CA* convert short reads into long sequences, and utilize overlap-layout-consensus (OLC) assemblers to finish the assembly, whereas the other assemblers assemble the short reads using de Bruijn graphs. The OLC approach appears to be superior to the de Bruijn graph approach in creating longer contigs.

**Table 1:** **Statistics of contigs assembled from simulated data**

Dataset ^a^	Program	Total length (bp)	Mean (bp)	N50 (bp)	N90 (bp)	Error ^b^
*D. melanogaster* (simulation)	*anytag* ^c^	113,166,478	66,141	197,693	43,974	109
	*ABySS* ^d^	116,966,148	5,795	177,493	33,254	89
	*MSR-CA*	116,924,670	48,396	163,131	34,562	346
	*soap* ^d^	113,971,825	16,208	56,061	13,361	90
	*velvet* ^d^	114,719,611	16,573	104,879	23,729	330
*Human Chr1* (simulation)	*anytag*	216,049,114	49,360	106,803	27,723	189
	*ABySS*	221,070,068	1,578	9,362	1,332	122
	*MSR-CA*	218,489,997	16,398	37,472	9,204	1,785
	*soap*	221,093,414	4,002	21,237	5,295	46
	*velvet*	Out of memory ^e^				

^a^All programs use the same simulated raw data. Our dataset was generated into four libraries, with insert sizes at 200 bp, 300 bp, 400 bp and 600 bp. Sequencing error was simulated at 0.005 and randomly distributed on the reads. The diploid heterozygosity is set at 0.001.

^b^Error = Inversion + Relocation + Translocation. The evaluation was completed by the evaluator from *GAGE*.

^c^ *anytag* constructed pseudo-Sanger sequences, *Newbler* and *minimus2* were used to assemble pseudo-Sanger sequences.

^d^kmer size was iteratively set to 21, 25, 31, 41, 51 for *ABySS*, *SOAPdenovo* and *velvet*. The assembly with the largest N50 contig was showed.

^e^Our memory limit is 450 G bytes.

We evaluated the large mis-assemblies (inversion, relocation and translocation) of all assemblies using evaluator from *GAGE*. Anytag introduced a bit more large mis-assemblies than short reads assemblers based on *De Bruijn* graph. *MSR-CA* got worst performance in evaluation. Both those two assemblers lies on third-party OLC assemblers. However, it is expected to validate and revise the contigs by paired-end reads mapping in genome assembly.

### Comparison of the pseudo-Sanger approach with other methods in assembling the drosophila genome from experimental data

We applied the pseudo-Sanger approach to the genome of the *w*^*1118*^ strain of *Drosophila melanogaster*. Unlike the reference strain *ISO-1*[15], this line is not isogenic and has an estimated heterozygosity of 0.328% per site in our sequencing data. This level of heterozygosity is less than half of the population genetic diversity of the species [16, 17]. As shown in Figure 1, the genomic DNA was randomly sheared into a series of decreasing fragment sizes and was used to create a nested set of paired-end libraries with insert sizes of 200 bp, 300 bp, 400 bp and 600 bp. All libraries were subjected to Illumina paired-end sequencing.

In total, 165.4 million 100 bp paired-end short reads were produced. Running *anytag* with 8 threads for 37.5 hours, we were able to construct 15.1 million (sum up to 8.8 G bases) pseudo-Sanger sequences with an average length of 581.41 bp. These long sequences cover the *D. melanogaster* genome at a depth of 55×. The assembly of pseudo-Sanger sequences yielded 2,307 contigs greater than 100 bp in length, and the N50 contig length was 190,040 bp.

We also investigated the best assembler using the *w*^*1118*^ dataset. In almost every category in Table 2, the pseudo-Sanger assembly performed substantially better than other methods. The pseudo-Sanger method produced similarly sized contigs on both the simulated and real datasets, but the performances of other assemblers often dropped sharply on the real data. In addition to comparing methods based on the same SRS platform, we also compared methods for a different data platform. For that purpose, we downloaded three 454 datasets from the Sequence Read Archive (SRA, SRX015853 ~3.4 G bases, SRX01-5856 ~3.0 G bases and SRX015861 ~3.0 G bases) of the Drosophila Genetic Reference Panel (DGRP) (http://dgrp.gnets.ncsu.edu). All 3 datasets are based on isogenic lines. The whole genome coverage is 19-21X, which is quite enough for isogenic genomes. Although the 454 platform generates long reads (> 400 bp) directly, the PS method compensates for its extra step by its error-correction and rare homo-polymer errors. It is also worth noting that 454 data costs much more to generate.

**Table 2:** **Statistics of contigs assembled from the experiment data from** ***D. melanogaster***

Dataset	Program	Total length	Mean	N50	N90
Illumina paired-end short reads from the nonisogenic *w* ^*1118*^ line	*anytag*	127,234,490	55,151	190,040	31,389
	*ABySS*	140,898,203	2,848	35,179	3,958
	*MSR-CA*	150,524,058	4,421	17,210	2,055
	*soap*	132,954,582	1,270	4,705	536
	*velvet*	Out of memory			
454 long reads from 3 isogenic lines ^a^	*Newbler*	123,157,508 ~ 128,620,384	5,197 ~ 6,896	33,241 ~ 75,801	2,367 ~ 5,621
20X PS reads ^b^	*anytag*	121,492,910	29,698	104,623	16,587

^a^NCBI SRA accessions: SRX015853, SRX015856 and SRX015861.

^b^20X PS reads were randomly selected to fit the coverage of 454 long reads.

In Table 2, 454 sequences were assembled using *Newbler*. The longest N50 contig length from the 454 data is 75,801 bp, much shorter than that from the pseudo-Sanger method. The mean contig length and N90 contig length are also both shorter by the 454 data. 20× PS reads were randomly selected and assembled to generate an equal coverage of 454 dataset. The reduced dataset produced N50 contig size of 104,623 bp.

### Evaluating the pseudo-Sanger approach on a large genome

Assembly of large genomes (such as the human genome) poses additional challenges. First, an assembler needs to handle billions of short reads in memory. Second, the increased computing time can become an important issue. Repetitive sequences also become a bigger concern in larger genomes. For example, *ABySS* performed surprisingly worse with our simulated human chromosome 1 dataset than with the Drosophila one.

The Naked Mole Rat (NMR) genome is about 2.74 Gb, and was firstly assembled using *SOAPdenovo*[18]. The NMR assembly project generated nested libraries with insert sizes of 170 bp, 350 bp, and 500 bp with some long inserts of up to 20 kb. Although such paired-end libraries are not ideal for the pseudo-Sanger method, they may still provide a crude glimpse of its effectiveness in assembling large genomes. We downloaded 1,199 mil-lion 100 bp paired-end reads from three libraries (170 bp, 350 bp and 500 bp). The raw Illumina reads cover the NMR genome with a depth of 12.0X, 14.9 ×, and 16.9×, respectively. Our program, *anytag*, ran for 62 hours on 8 cores and constructed 122.3 million pseudo-Sanger sequences with a mean length of 442 bp. Using *Newbler* and *minimus2*-blat [13] to assemble the long sequences, we obtained the initial contigs with a mean of 12.3 kb, an N50 of 23.3 kb, and an N90 of 6.2 kb. We also tried other assemblers on the same dataset, but only *SOAPdenovo* finish the assembly. *ABySS* ran out of 450G memory. *MSR-CA* ran out of time (two weeks’ limit). *SOAPdenovo* obtained the best assembly under k-mer size of 31, its N50 contig size was 14,441 bp, N90 contig size was 3,016 bp. Please note that we did not perform either scaffolding with the long jump libraries or filling gaps (which always increases the contig length largely), but our contigs are even better than the published NMR assembly [18] with long jump libraries (N50 = 19.3 k, N90 = 4.7 k).

## Discussion

We presented the Pseudo-Sanger sequencing (PS) method to produce long and near error-free sequences with high throughput by filling the gaps between the paired-end short reads produced by NGS platforms. Compare to other gap-filling method, the PS method presented advantages on the aspects of read accuracy and repeat tolerance.

One obvious advantage of the PS method is that the error rate of the produced long sequences is extremely low. By local assembly of highly redundant reads, almost all the errors in the original short reads were corrected with very few remained in constructed long reads, and the allele of heterozygote with relatively higher frequency is kept as reference allele. In our simulation, the assembly of less repetitive genome was improved significantly due to error correction and heterozygote merge.

Another advantage of pseudo-Sanger method is that the structure of repetitive elements shorter than the insert size of anchor reads (~600 bp in the case) can be solved spontaneously. We used both end of anchor reads as a tag, supporting reads have one end match the tag are clustered to do local assembly. This strategy is like SubAssembly [11], which uses one of paired-end reads as tag, and local assembly of the other ends to build a contig of long DNA fragment. Nevertheless, our two short reads tag has more sensitivity and specificity over SubAssembly’s 17-base single end tag. Even when our two paired reads tag are repetitive, we are able to correctly recover full-length sequences theoretically by carefully examining the multiple paths connecting two ends of AR. However, when both ends of AR lie in highly repetitive regions, there will be too many SRs involved in the local assembly process, which makes local assembly extremely slow and it is difficult to distinguish the correct PS sequences from thousands of possible paths. In practice, we calculate the repetitiveness of ARs and refuse to do local assembly on highly repetitive regions.

In comparison with other methods of long read construction [5–8], the PS method takes advantages of the length space of the genomic fragments and generates long reads about five times longer than the original short reads, which outperforms the existing methods. For example, *SHERA*[5], *FLASH*[6] and *COPE*[7] could at most double the length of single short reads by identifying the overlapped part of paired reads sequenced from short DNA fragments. Although *GapFiller* produces long reads up to 3.5 kb in size [8], it can hardly resolve repeats, which largely restricts its application in large eukaryotic genomes.

Continuous insert sizes libraries are upmost ideal for pseudo-sanger method. However, the cost of library construction should be in consideration. For small genomes, at least two libraries must be provided (Additional file 1: Note S1).

The potential applications of the PS method are extensive. Because of the possibility of routine usage, most problems that require long and error-free sequences in high throughput can benefit from this method. We chose de novo genome assembly for a demonstration. Although next generation sequencing (NGS) techniques have been used successfully to assemble large genomes [19], the direct de novo assembly of SRS often leaves many gaps in the scaffolds and assemblies of questionable quality [3]. By first converting short reads into pseudo-Sanger sequences, we show that whole genome assembly using NGS sequencing platforms can be done efficiently. The contigs generated from the PS sequences are much longer than from SRS directly (Table 2). It can be reasoned that longer contigs would generate longer scaffolds if given long jump reads, and thus contribute to better genome assembly. Interestingly, our results were at least as good, if not slightly better, than assemblies based on 454 sequencing but come at a fraction of the cost.

The pseudo-Sanger method is a general approach that fills in the sequence between paired-end or mated-pair reads. Because the expected number of SRs for one AR is linearly correlated with read length times sequencing depth, PS sequences longer than the Sanger-sequence length have not been practical to obtain. Now that paired reads are becoming much longer, the distance between the pairs can be increased correspondingly. The resulting pseudo-Sanger (or super-Sanger) sequences of a few kb with errors corrected may greatly expand the general utility of NGS sequencing.

## Conclusions

By paired-end sequencing of a series of stepwise insert size libraries, we are able to recover the full length sequences of the largest DNA fragments using computational method. Smaller DNA fragments are aligned to the largest DNA fragments by one of their two-ends. Thus, the other ends can be used to fill the un-sequenced regions in the largest DNA fragments. Our local assembly enable to remove partial matched DNA fragments (small repeats), correct sequencing errors, and tolerate heterozygote. By recovering full length sequences of paired-end sequencing, de novo assembly can be improved significantly. Besides, PS sequences can be applied for many other problems requiring long DNA sequences, such as the detection of structural variations and the analysis of metagenomics diversity.

## Methods

### Evaluation of pseudo-Sanger sequencing

We performed pseudo-Sanger sequencing on both simulated and experimental data. In the simulations, a series of libraries with stepwise decreasing insert size were generated using a modified version of *wgsim* (https://github.com/lh3/wgsim). We then employed a two-step process: 1) assembly of pseudo-Sanger sequences using our software *anytag* and 2) whole genome assembly into contigs using a long reads assembler. As a proof of concept, we also sequenced the *Drosophila melanogaster* line *w*^*1118*^. Comparisons of contigs were carried out between the pseudo-Sanger method and other software and sequencing platforms. For short read assembly, we tested against *velvet*[20], *ABySS*[21], *MSR-CA* (ftp://ftp.genome.umd.edu/pub/MSR-CA/) and *SOAPdenovo*[22] using the same dataset. For long read assembly, we used publicly available 454 reads in our comparisons. Besides basic contig statistics (total length, mean, N50, and N90), we also evaluated the accuracy of the contigs. We also evaluated the performance of pseudo-Sanger sequencing on a large genome (2.74 Gb), the Naked Mole Rat genome. A detailed evaluation can be found in the supplementary material (Additional file 1).

### Simulation of reads

*wgsim* from *Samtools*[23] was modified to simulate data with a wide-range of insert sizes and various levels of heterozygosity (http://sourceforge.net/projects/anytag/files/). The simulated reads contained random sequencing error uniformly distributed across the read. We did not simulate genomic coverage bias or chimeric reads.

### Pseudo-Sanger assembly

A series of paired-end read libraries with stepwise decreasing insert sizes were indexed using blocked spaced seeds. The paired reads from the largest insert size library (ARs) were then used to query the spaced seed index to find all possible overlapping SRs from the smaller insert size libraries. For each AR, a localized group of SRs were found. Next, a local assembly was performed to build a consensus sequence for each AR.

### Long reads assembly

Pseudo-Sanger sequences and 454 reads were assembled using *Newbler*. The general parameters used in this study were “-large –m –nobig –noace –cou 16”. “-het” was added in assembly of both the simulated data and the short read of the non-isogentic line *w*^*1118*^. If pseudo-Sanger sequences cover the genome at greater than 20X coverage, we shuffle the pseudo-Sanger sequences into multiple groups (each about 8X). *Newbler* was then used to assemble the small parts into contig sets. *Minimus2* was used to get the consensus contigs. When the genome size is big, such as with human chromosome1 or Naked Mole Rat, *minimus2-blat* was used instead of *minimus2*.

### Short sequence reads assembly

*MSR-CA* was executed with default parameters except the JF_SIZE value was set to be large enough for jellyfish. For *ABySS*, *velvet* and *SOAPdenovo*, the k-mer size was iteratively selected from 21, 25, 31, 41, and 51. The special parameters in velvet were “-exp_cov 60 –cov_cutoff auto”. The special parameters in *SOAPdenovo* were “-M 3 -d 2 -D 2 -R -F”. We used substring scaffolds without any N (the arbitrary base) as contigs.

### Library construction

Here we outline the experimental procedures. Genomic DNA was extracted using Phenol-Chloroform from freshly frozen Drosophila melanogaster of the line *w*^*1118*^ and subsequently sonicated to create fragments ranging from 200–600 bp in size. Multiple size selections were performed using electrophoresis, and bands corresponding to sizes of 200, 300, 400, and 600 bp were excised and purified from a single continuous DNA smear. Each group of size-selected fragments was then blunted, A-tailed, and ligated to Illumina Paired-End adaptors. A second round of size selection was performed on each group of adaptor-ligated libraries, and target fragments with the added adaptors were chosen. 9–12 cycles of PCR amplification were then performed with standard Illumina primers on each group of libraries. After PCR amplification, a third round of size selection was conducted to extract amplified target segments and remove redundant fragments such as PCR primers. After PCR amplification, each sub-library was then quantified using *Qubit* qPCR and subsequently size-validated using Agilent *Bioanalyzer 2100*.

### Illumina sequencing

Each of the libraries in the series of insert sizes was treated as a standard library, and their respective sizes were used for calculating the molar mass needed for cluster generation. 100 × 100 bp paired-end reads were generated using an Illumina *HiSeq2000* instrument according to the manufacturer’s standard specifications.

### Sequencing error estimation

The alignment program *BWA*[24] was used to directly map the short reads while *BWA-SW*[25] was used to map the longer pseudo-Sanger and Sanger reads back to the reference genome dm3, using default parameters. SAM files from bwa or bwasw were then processed by *SAMtools*[23] for further analysis.

### Estimation of *w*^*1118*^’s heterozygosity

*SAMtools* was used to pileup sequences along the reference genome dm3. The variants calling parameter from the short reads alignment was set as “samtools mpileup –C50 –E –u” to generate a BCF file. Then “vcfutils.pl varFilter –D200” was run to generate a VCF file of final variants. A SNP dataset called from the short reads was regarded as *w*^*1118*^’s germline polymorphisms. The heterozygosity of the *w*^*1118*^ line was also estimated using this information.

### Evaluation of contigs

Contigs were evaluated by evaluator from *GAGE*[14]. Large mis-assemblies including Inversions, relocations and translocations were summed as assembly errors.

### Availability of supporting data

The raw Illumina sequence reads used in this study have been submitted to the NCBI Short Reads Archive (http://www.ncbi.nlm.nih.gov/sra) under accession number SRA101397. The raw Illumina sequence reads, pseudo-Sanger sequences, 454 reads, and genome assemblies are also freely available at a public FTP server ftp://ftp.big.ac.cn/pub/pseudo-sanger-demo/. Software implemented for this approach is available at http://sourceforge.net/projects/anytag/files/.

## Electronic supplementary material

Additional file 1: **Note S1.** How to prepare libraries for Pseudo-Sanger. **Note S2.** Assembling pseudo-sanger sequences by *Newbler* and *minimus2.* **Table S1.** Statistics on the assembly of *Drosophila melanogaster* genome using simulated reads. **Table S2.** Statistics on the assembly of human chromosome 1 using simulated reads. **Table S3.** Statistics on the assembly of *D. melanogaster w* ^*1118*^ using experimental data. **Table S4.** Statistics on the assembly of Naked Mole Rat using read data. **Figure S1.** Base error rate distribution along the positions on short and pseudo-Sanger reads. **Figure S2.** Electrophoresis image for fragment lengths. **Figure S3.** Library insert sizes inferred from mapping results. **Figure S4.** Tests of various library inserts sequenced in a single lane. (DOC 340 KB)
