# Supplementary material for: Pseudo-Sanger sequencing: massively parallel production of long and near error-free reads using NGS technology
Source: BMC Genomics. 2013 Oct 17;14(1):711. doi: 10.1186/1471-2164-14-711 (PMC4046676; doi:10.1186/1471-2164-14-711)
Supplement: Supplementary file 1 — Additional file 1: Note S1. How to prepare libraries for Pseudo-Sanger. Note S2. Assembling pseudo-sanger sequences by Newbler and minimus2. Table S1. Statistics on the assembly of Drosophila melanogaster genome using simulated reads. Table S2. Statistics on the assembly of human chromosome 1 using simulated reads. Table S3. Statistics on the assembly of D. melanogaster w 1118 using experimental data. Table S4. Statistics on the assembly of Naked Mole Rat using read data. Figure S1. Base error rate distribution along the positions on short and pseudo-Sanger reads. Figure S2. Electrophoresis image for fragment lengths. Figure S3. Library insert sizes inferred from mapping results. Figure S4. Tests of various library inserts sequenced in a single lane. (DOC 340 KB) [file 12864_2013_5437_MOESM1_ESM.doc]

# Supplementary Notes

## Note S1 How to prepare libraries for Pseudo-Sanger

Pseudo-Sanger method requires a nested of paired-end libraries. Typically, for 2X100bp PE sequencing, the insert sizes of libraries are 200bp, 300bp, 400bp, and 600bp. Usually, we do not put libraries with different insert sizes into one lane of *Illumina* sequencer. Therefore, for small genomes, four lanes of sequences will be redundant. In our experience from early stage of development (See Supplementary Figure S2-4), to pool different insert size libraries into one *Illumina GAII* lane, the DNA input (mol) of the relative larger should be 15-20% more than the smaller.

The number of the libraries can be flexibly adjusted depending on different genomes. Besides 4 libraries as used in our presented work, Pseudo-Sanger also worked well with two libraries (200 bp and 500bp), which was useful for small genomes such as rice and fly (Tested by the authors). We also tested five libraries (+800bp) in the assembly of wolf genome; Pseudo-Sanger produced much more excellent contigs than *SOAPdenovo*. As the read length increases, taking 2X150bp PE for instance, the insert sizes could be 250bp, 500bp and 700bp (Untested).

## Note S2 Assembling pseudo-sanger sequences by *Newbler* and *minimus2*

When the pseudo-sanger sequences cover the genome no more than 16X, we directly assembled them using *Newbler*. For deeper coverage, we first split pseudo-sanger sequences into many parts of which about 8X, and assemble them one by one using *Newbler*. Then, *minimus2* was used to merge the first assembly and second assembly, the merged assembly was next merged with third assembly, and so on. If the genome is very big (more than 200M), *minimus2* will be very slow, *minimus2*-blat is used to finish the merging quickly.

# Supplementary Tables

**Table S1 Statistics on the assembly of *Drosophila melanogaster* genome using simulated reads**

| **Software** | **kmer size** | **Total Length** | **Mean** | **N50** | **N90** | **Error** |
| --- | --- | --- | --- | --- | --- | --- |
| ***SOAPdenovo*** | 21 | 113971825 | 16207 | 56061 | 13361 | 90 |
| ***SOAPdenovo*** | 25 | 114148373 | 15989 | 52197 | 12830 | 90 |
| ***SOAPdenovo*** | 31 | 114419945 | 14583 | 44062 | 11424 | 69 |
| ***SOAPdenovo*** | 41 | 114872492 | 11940 | 35837 | 9804 | 21 |
| ***SOAPdenovo*** | 51 | 117657518 | 3628 | 31971 | 8045 | 11 |
| ***ABySS*** | 21 | 112308420 | 1067 | 2828 | 461 | 58 |
| ***ABySS*** | 25 | 114084371 | 4879 | 15361 | 2953 | 74 |
| ***ABySS*** | 31 | 114227755 | 14707 | 97710 | 17673 | 89 |
| ***ABySS*** | 41 | 114905906 | 17996 | 169915 | 34121 | 82 |
| ***ABySS*** | 51 | 116966148 | 5794 | 177493 | 33254 | 89 |
| ***velvet*** | 21 | 114103984 | 1284 | 2272 | 619 | 3303 |
| ***velvet*** | 25 | 114215722 | 5640 | 14324 | 3175 | 753 |
| ***velvet*** | 31 | 113893688 | 12642 | 51685 | 10882 | 383 |
| ***velvet*** | 41 | 114328544 | 16895 | 96636 | 21303 | 317 |
| ***velvet*** | 51 | 114719611 | 16573 | 104879 | 23729 | 330 |
| ***MSR-CA*** | - | 116924670 | 48396 | 163131 | 34562 | 346 |
| ***anytag*** | - | 113166478 | 66141 | 197693 | 43974 | 109 |

**Table S**2 Statistics on the assembly of human chromosome 1 using simulated reads

| **Software** | **kmer size** | **Total Length** | **Mean** | **N50** | **N90** | **Error** |
| --- | --- | --- | --- | --- | --- | --- |
| ***SOAPdenovo*** | 21 | 207264080 | 5468 | 12639 | 3123 | 146 |
| ***SOAPdenovo*** | 25 | 209526982 | 5958 | 14473 | 3540 | 158 |
| ***SOAPdenovo*** | 31 | 210763843 | 5374 | 13400 | 3183 | 113 |
| ***SOAPdenovo*** | 41 | 213785837 | 4804 | 12254 | 3025 | 83 |
| ***SOAPdenovo*** | 51 | 221093414 | 4002 | 21237 | 5295 | 46 |
| ***ABySS*** | 21 | 158585670 | 538 | 1195 | 169 | 110 |
| ***ABySS*** | 25 | 176005964 | 972 | 2463 | 437 | 169 |
| ***ABySS*** | 31 | 189999203 | 1158 | 3327 | 567 | 167 |
| ***ABySS*** | 41 | 207418174 | 1403 | 5154 | 799 | 153 |
| ***ABySS*** | 51 | 221070068 | 1578 | 9362 | 1332 | 122 |
| ***MSR-CA*** | - | 218489997 | 16398 | 37472 | 9204 | 1785 |
| ***anytag*** | - | 216049114 | 49360 | 106803 | 27723 | 189 |

**Table S3 Statistics on the assembly of *D. melanogaster w^1118^* using experimental data**

| **Software** | **kmer size** | **Total Length** | **Mean** | **N50** | **N90** |
| --- | --- | --- | --- | --- | --- |
| ***SOAPdenovo*** | 21 | 132954582 | 1270 | 4705 | 536 |
| ***SOAPdenovo*** | 25 | 135497398 | 1217 | 4011 | 520 |
| ***SOAPdenovo*** | 31 | 138279305 | 1173 | 3623 | 503 |
| ***SOAPdenovo*** | 41 | 143964164 | 1082 | 3228 | 416 |
| ***SOAPdenovo*** | 51 | 151561604 | 960 | 2932 | 292 |
| ***ABySS*** | 21 | 114827424 | 765 | 2025 | 236 |
| ***ABySS*** | 25 | 119868876 | 2383 | 9214 | 1608 |
| ***ABySS*** | 31 | 125614476 | 3533 | 30803 | 4341 |
| ***ABySS*** | 41 | 140898203 | 2848 | 35179 | 3958 |
| ***ABySS*** | 51 | 166365232 | 1416 | 26916 | 2114 |
| ***MSR-CA*** | - | 150524058 | 4421 | 17210 | 2055 |
| ***anytag*** | - | 127234490 | 55151 | 190040 | 31389 |

**Table S4 Statistics on the assembly of Naked Mole Rat using read data**

| **Software** | **kmer size** | **Total Length** | **Mean** | **N50** | **N90** |
| --- | --- | --- | --- | --- | --- |
| ***SOAPdenovo*** | 21 | 2116289904 | 4455 | 10975 | 2667 |
| ***SOAPdenovo*** | 25 | 2168516731 | 4720 | 12958 | 2987 |
| ***SOAPdenovo*** | 31 | 2226892257 | 4364 | 14441 | 3016 |
| ***SOAPdenovo*** | 41 | 2306682205 | 3488 | 14001 | 2518 |
| ***SOAPdenovo*** | 51 | 2422889901 | 2272 | 13665 | 1938 |
| ***ABySS*** | 21 | Out of memory | | | |
| ***ABySS*** | 25 |
| ***ABySS*** | 31 |
| ***ABySS*** | 41 |
| ***ABySS*** | 51 |
| ***MSR-CA*** | - | Out of time limit (two weeks) | | | |
| ***anytag*** | - | 2135618892 | 12325 | 23276 | 6232 |
|  |  |  |  |  |  |

# Supplementary Figures


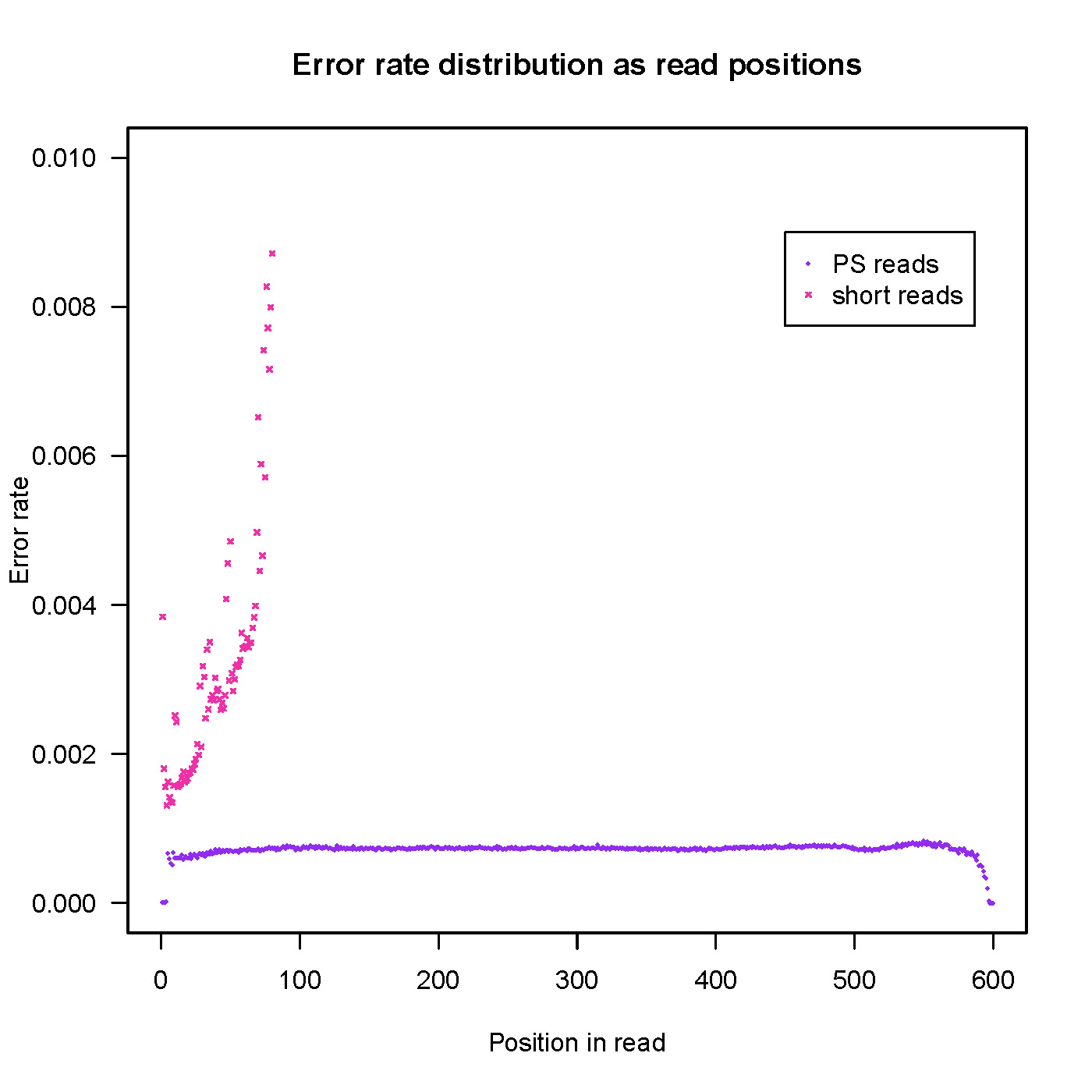


**Figure S1 Base error rate distribution along the positions on short and pseudo-Sanger reads.**


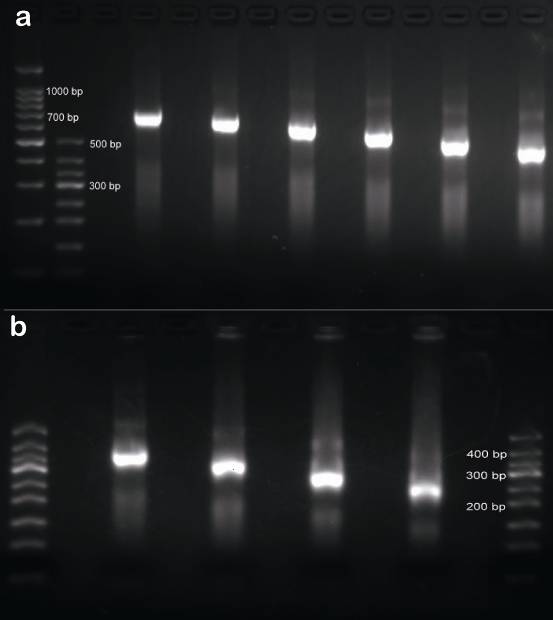


**Figure S2. Electrophoresis image for fragment lengths**

Size selection of adapter-ligated fragments from ten sub-libraries was performed using 3% argarose gel**.**


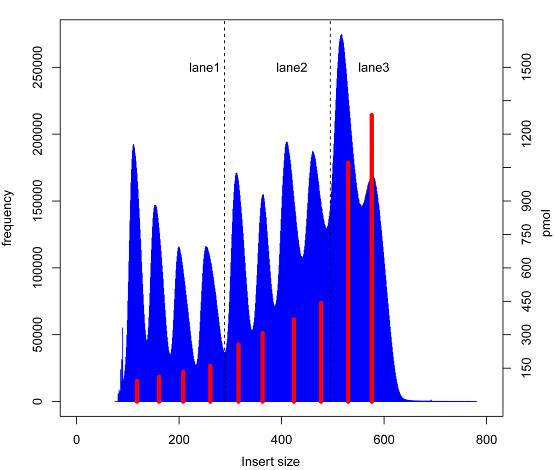


**Figure S3. Library insert sizes inferred from mapping results**

10 sub-libraries were quantified and mixed into three spread-size libraries; insert size of 100-300 bp, 300-500bp and 500-600bp. Each spread-size library was sequenced with an *Illumina GA-II* Paired-end lane. The height of each red bar represents DNA content (in pmols) of individual library before cluster generation. The x axis indicates the size of each sub-libraries measured using *Agilent* *Bioanalyzer* *2100*. After sequence reads being mapped to the reference genome with *BWA*, density plot for the observed insert size from the mapped paired-end reads are shown in blue.


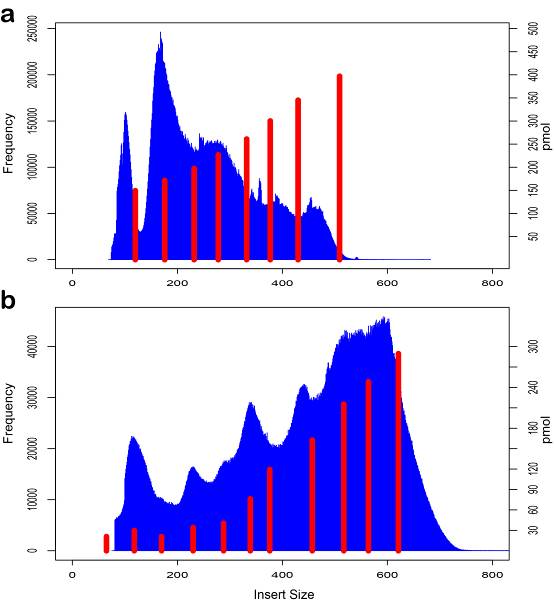


**Figure S4. Tests of various library inserts sequenced in a single lane**

**a)** Test 1: a spread-size library with insert size ranging from 100 to 500 bp with a single *Illumina GAII* lane. 8 sub-libraries were mixed with an increasing molar mass of 15% every time the insert size increase by 50bp (red bars). Based on the mapping result of the data, large fragments were under-represented (blue density plots). **b)** In the Test 2: Insert size ranged from 100 to 600 bp with a single lane was conducted. 10 sub-libraries were mixed with larger molar mass increase (more than 20%) when the average insert size of sub-libraries increased by 50 bp. Based on the mapping result, small fragments were under-represented in this case (blue density plot).
